# Investigating people’s attitudes towards participating in longitudinal health research: an intersectionality-informed perspective

**DOI:** 10.1186/s12939-022-01807-0

**Published:** 2023-01-31

**Authors:** Sibille Merz, Philipp Jaehn, Tobias Pischon, Beate Fischer, Kerstin Wirkner, Stefan Rach, Kathrin Guenther, Nadia Obi, Christine Holmberg, the AdvanceGender Study Group, Gabriele Bolte, Emily Mena, Alexander Rommel, Anke-Christine Saß, Kathleen Pöge, Sarah Strasser

**Affiliations:** 1 Institute of Social Medicine and Epidemiology, Brandenburg Medical School Theodor Fontane, Hochstr. 15, 14770 Brandenburg an der Havel, Germany; 2 Faculty of Health Sciences, joint Faculty of the Brandenburg University of Technology Cottbus – Senftenberg, Brandenburg Medical School, University of Potsdam, Fehrbelliner Str. 38, 16816 Neuruppin, Germany; 3 Max-Delbrueck-Center for Molecular Medicine in the Helmholtz Association (MDC), Molecular Epidemiology Research Group, Robert-Rössle-Straße 10, 13125 Berlin, Germany; 4 Max-Delbrueck-Center for Molecular Medicine in the Helmholtz Association (MDC), Biobank Technology Platform, Robert-Rössle-Straße 10, 13125 Berlin, Germany; 5 Berlin Institute of Health at Charité - Universitätsmedizin Berlin, Core Facility Biobank, Anna-Louisa-Karsch-Straße 2, 10178 Berlin, Germany; 6 Charité - Universitätsmedizin Berlin, corporate member of Freie Universität Berlin and Humboldt-Universität zu Berlin, Charitépl. 1, 10117 Berlin, Germany; 7 University of Regensburg, Department of Epidemiology and Preventive Medicine, Franz-Josef-Strauß-Allee 11, 93053 Regensburg, Germany; 8 LIFE – Leipzig Research Centre for Civilization Diseases, Philipp-Rosenthal-Straße 27, 04103 Leipzig, Germany; 9 Leibniz Institute for Prevention Research and Epidemiology - BIPS, Achterstraße 30, 28359 Bremen, Germany; 10 University Medical Center Hamburg-Eppendorf, Martinistraße 52, 20251 Hamburg, Germany

## Abstract

**Background:**

Increasing evidence suggests that participation proportions in longitudinal health research vary according to sex/gender, age, social class, or migration status. Intersectionality scholarship purports that such social categories cannot be understood in isolation and makes visible the co-dependent nature of the social determinants of health and illness. This paper uses an intersectionality-informed approach in order to expand the understanding of why people participate in health research, and the impact of intersecting social structures and experiences on these attitudes.

**Methods:**

A sample of 80 respondents who had previously either accepted or declined an invitation to participate in the German National Cohort (NAKO) participated in our interview study. Interviews were semi-structured and contained both narrative elements and more structured probes. Data analysis proceeded in two steps: first, the entire data set was analysed thematically (separately for participants and non-participants); second, key themes were compared across self-reported sex/gender, age group and migration status to identify differences and commonalities.

**Results:**

Respondents’ attitudes towards study participation can be categorised into four themes: wanting to make a contribution, seeking personalised health information, excitement and feeling chosen, and seeking social recognition. Besides citing logistical challenges, non-participants narrated adverse experiences with or attitudes towards science and the healthcare system that deterred them from participating. A range of social experiences and cultural value systems shaped such attitudes; in particular, this includes the cultural authority of science as an arbiter of social questions, transgressing social categories and experiences of marginalisation. Care responsibilities, predominantly borne by female respondents, also impacted upon the decision to take part in NAKO.

**Discussion:**

Our findings suggest that for participants, health research constitutes a site of distinction in the sense of making a difference and being distinct or distinguishable, whereas non-participants inhabited an orientation towards science that reflected their subjective marginalisation through science. No clear relationship can thereby be presumed between social location and a particular attitude towards study participation; rather, such attitudes transgress and challenge categorical boundaries. This challenges the understanding of particular populations as more or less disadvantaged, or as more or less inclined to participate in health research.

## Introduction

Understanding participants’ motives to join longitudinal health research is crucial to stimulate participation and retention [19, 49]. Previous research has illustrated that participants enrol in health research due to a complex variety of individual and institutional factors, study-related aspects and broader social dynamics [55, 56, 33, 6, 29, 34, 60]. Attitudes towards research participation have been found to be multi-faceted, complex and often profoundly social in origin rather than based on rational, calculated, individual decision-making [29, 33, 6, 60]. Participation proportions also vary considerably across social groups [1, 23, 63]. For instance, participation proportions in surveys and genomic cohort studies have been found to be lower for men [47] and ethnic minorities [44,61,37,20] or migrant populations [63,74]. Participants in longitudinal research are more likely to be educated to at least undergraduate level [11,25]. In cohort studies, women aged 75 and older and women aged 35 and younger are often underrepresented groups amongst women [35], and the influence of a steady partnership on response proportions differs by sex/gender [66]. However, most analyses of social heterogeneity have either exclusively focused on specific subgroups [61,67,4,37,42] or have been quantitative and descriptive in nature, missing to engage the complex and situated nature of study participation (also see [29]. Intersectionality scholarship has focused on the interactions between different social categories and the structural causes they are proxies for, shaping individual experience and (health) inequities [14,17,15]. For instance, Kimberlé Crenshaw [17], who initially coined the term intersectionality, detailed the experience of Black women in the US, discriminated against by both sexism and racism simultaneously. She uses the analogy of traffic at an intersection that is coming and going in all directions; like traffic, discrimination may flow in one direction or another and when an accident happens, it can be caused by one car or another, or by several cars simultaneously. Similarly, if Black women are harmed, this can be the result of racism or sexism – or both. Intersectionality thereby foregrounds the interaction of different social locations at the individual level but also lends greater attention to the macro causes determining social inequity, aiming to further the understanding of but also to dismantle interlocking systems of oppression and privilege. That is, rather than examining social identities, intersectional approaches focus on the power dynamics and structural causes that determine them, for example racism, sexism, classism etc. This is crucial as the ultimate objective is to deconstruct barriers to the realisation of social equity and justice. Intersectional perspectives thereby go beyond purely additive approaches that consider multiple factors, often positing that social inequity increases with each added marginalised identity [8]. Such approaches have been aptly criticised as they conceptualise people’s complex social experiences as separate, independent and summative. Instead, intersectional approaches focus on the unique experiences conferred by the interaction of multiple factors. For instance, in Crenshaw’s original case, the experience of Black women cannot be explained by examining the effects of racism and sexism in isolation; their experience is above and beyond being Black or female [17]. Moreover, intersectional approaches challenge the social determinants of health (SDH) framework that is often overly deterministic and tends to omit in-group variations [52]. While SDH perspectives have been crucial for rendering existing health inequities visible, they often lack a comprehensive conceptualisation of the complexity of the societal causes underlying health inequity, and the dynamic nature of social relations and experience. Not least, unlike intersectional perspectives, SDH frameworks have been accused of eschewing radical claims towards social transformation and the redistribution of power and material resources (ibid).

While the interest in intersectional perspectives in public health has increased over the last few years [2, 9, 10, 26, 30, 41, 52, 51, 53, 39], the framework has not been comprehensively applied to advance the understanding of differential participation in longitudinal health research. In past discourses on recruitment and representativeness in clinical and public health research, especially in relation to the mandate set out by the US-based National Institutes of Health (NIH), the focus has predominantly been on the inclusion of ‘women and minorities’ [22, 9], neglecting the fact that populations are not mutually exclusive but rather interacting and multidimensional. Intersectionality scholars have argued for the need to build in complexity in recruitment and sampling strategies rather than treating intersecting social locations as static, homogeneous and distinct social categories [9].

In this paper we thus employ an intersectionality-informed perspective to investigate how attitudes towards participating in cohort studies are shaped by individuals’ multiple and intersecting social locations in a sample of people who had been invited to participate in the German National Cohort (*NAKO Gesundheitsstudie*, abbr. NAKO), Germany’s largest prospective cohort study that aims to investigate the causes of major chronic diseases [24, 45]. During the NAKO baseline examinations running from 2014 to 2019, a random sample of the general population consisting of 205,415 women and men aged 19–74 years was examined in 18 regional study centres across Germany. Assessments included a wide range of medical examinations, the collection of various biomaterials, an extensive interview and self-completion questionnaires (see [57] for a comprehensive description). Potential NAKO participants were randomly drawn from regional registries of residents and were recruited according to a standard recruitment protocol (see [46] for details) that included an invitation letter, followed by up to three reminder letters, each separated by waiting periods of 14 days. For potential participants with known phone numbers, phone calls were attempted prior to sending reminder letters. The overall NAKO response at baseline was 17%.

Our own conceptual approach to intersectionality is thereby primarily anti-categorical (McCall, 2005) as it aims to deconstruct analytical categories that are often taken for granted. McCall distinguishes between such anti-categorical approaches, inter-categorical approaches that adopt, albeit only provisionally, existing analytical categories to document relations between them, and intra-categorical approaches that explore particular social groups at neglected points of intersection. While we used some categories, i.e. age and sex/gender, in our sampling process due to prior evidence about their potential significance in contemporary Germany (see Limitations), we did not assume these to be a priori significant during data analysis. An anti-categorical approach is analytically productive because it does not essentialise specific categories and allows for new or unexpected categories to emerge during analysis.

We also follow practice- or process-oriented approaches [12] in that we do not explore particular social identities or groups but apply an intersectional lens to study particular processes and situated practices, i.e. study participation, focussing on the “context and comparison at the intersections as revealing structural processes organising power” [12]: 134). The focus on practices counters the individualising features that characterises much quantitative (and some qualitative) health research, and resists the idea of causal relationships between social location and study participation by considering a range of material, cultural and social dimensions on which study participation is contingent [13]. To wit, by cultural dimensions we do not refer to a static set of characteristics or beliefs inhabited by particular groups but rather the dynamic, changing and transient systems of value and practices of meaning-making that underlie *all* human behaviour, albeit often unexamined [54]. Moreover, a qualitative, intersectionality-informed approach to the study of research participation that takes into account the limitations of categorical approaches to social life can capture how systems of oppression and privilege manifest in everyday practices, and how they intersect, rub against and perhaps even sit in tension with cultural value systems and personal experiences of health and illness. Not least, such an anti-categorical stance can uncover new dimensions of social heterogeneity at intersections previously unexamined or unexpected.

## Methods

### Study objectives and design

This paper emerges from AdvanceGender, a mixed-methods study developing a toolkit for gender-sensitive and intersectionality-informed methods for public health research and reporting. As part of this, the qualitative interview study with participants and non-participants in NAKO aimed to understand why (or why not) individuals participated in NAKO, what influenced this decision and, especially pertinent for this particular paper, how intersecting social locations may shape the decision-making process. A purposeful sampling strategy was developed with specific attention to participation, geographical location, sex/gender and age. A prospective sample of around 80 respondents (50 NAKO participants, 30 NAKO non-participants) was aimed at, with equal representation of men and women (40 each) and at least 20 respondents aged < 40. The larger sample size of NAKO participants was geared towards for two reasons: first, our research interest was to gain deeper knowledge about study participation as a complex practice shaped by multiple social dynamics [34, 6, 60]. While most epidemiological research has focused on reasons *against* participation, undoubtedly an important endeavour to improve the inclusion of underrepresented groups, the rationale of the larger project from which this paper stems aimed to examine the broader context in which study participation is embedded. From a societal perspective, study participation is much more unusual than non-participation [34] – most people do *not* participate in research, making participation a perplexing phenomenon for social science research. As such, the prime focus has been with those who actively decided to participate in NAKO. And second, the specific recruitment strategies available to the research team (i.e. sending a limited number of written invitations letters) were unlikely to yield a larger sample of non-participants.

### Recruitment and data collection

Respondents were recruited at five different NAKO study centres situated across Germany between March 2019 and June 2021. They were eligible to participate in the study provided that they had been invited to participate in NAKO and either accepted or declined this invitation. NAKO had invited a random sample of German residents aged 19–74 to participate [57]. The study centres were responsible for recruitment from their databases according to the sampling criteria laid out above. Precise strategies varied according to the specific data infrastructures of each study centre; while some drew the sample for our study from the overall samples of NAKO participants and non-participants, others drew this sample from pre-selected groups of participants and non-participants according to sex/gender and age group. In order to reach an overall sample of 80 respondents, assuming a relatively low response rate especially for non-participants, each study centre approached 250 individuals (80 NAKO participants and 170 non-participants) through formal letters of invitation that included contact forms and pre-paid and self-addressed envelopes. An exception was one study centre which was still conducting baseline assessment during recruitment and thus approached participants in person; non-responders were invited via post. Upon providing their contact details, potential respondents were contacted by the first author to arrange an appointment for the interview.

For the purpose of this paper we will use the terms ‘participants’ for those who had (at least) participated in the NAKO baseline assessment, ‘non-participants’ for those who had declined the invitation to participate in NAKO altogether, and ‘respondents’ for our own interviewees, both NAKO participants and non-participants.

### Qualitative interviews

Interviews were conducted in German by SM at locations preferred by participants, usually at their homes, or via telephone/video conference. The interviewer’s disciplinary background as a sociologist and her social positionality as a white, female, researcher with a PhD is likely to have influenced what some respondents were willing to share; existing reseach has shown that perceived social distance between interviewer and respondent based on characterstics such as age, gender or ethnicity can significantly affect data, especially of sensitive topics (e.g. [31]). Moreover, some interviews took place at the cooperating NAKO study centres, perhaps creating the perception that NAKO and our interview study were allied and thus eliciting more positive assessments of NAKO by respondents. SM and the research team reflected throughout the research on the impact of their social location on the data generated and the societal power relations imbricated in the researcher-participant relationship during regular regular data sessions, recording such reflections in memos (see Data Analysis).

Informed consent was obtained in written form before the interview. Interviews were conceptualised as semi-structured [58], containing narrative elements to stimulate conversation as well as more structured questions probing for details or additional dimensions of a particular question. Interview questions used for this paper addressed both respondents’ rationales for participation or non-participation and their experiences at the study centre, as well as their overall social experiences of inclusion and marginalisation as outlined in Table 1 Questions addressing discrimination and overall experiences were framed in an open-ended manner without reference to a particular dimension of respondents’ social locations to invite respondents to stress those aspects (most) meaningful to them [8]. Interviews were recorded on an audio device, and interview logs were written immediately after to record the interviewer’s initial observations. Interviews lasted between 19 and 140 min.

**Table 1:** Sample questions

Theme	Sample question(s)	Follow-up questions
Opening question	Could you please tell me a bit more about what made you participate in the study (or not), and what kind of experiences you have made so far?	
Decision-making process and experiences with the study	Could you please describe the situation in which you received the invitation letter?	Did you discuss your decision (not) to participate with anyone?
	Which expectations did you have vis-à-vis the study?	Have your expectations been met? Why (not)?
	How would you describe your overall experiences with the study?	Is there anything you remember particularly well?
	Are you planning on remaining in the study? Why (not)? Would you participate in a different study?	Why (not)? Why (not)? Why was that?
Experiences with the healthcare system	How would you describe your overall experiences with the healthcare system, for example when visiting your doctor?	
Experiences of marginalisation and/or privilege	Have there been situations in your life, for example in school or on the labour market, where you felt you had been socially disadvantaged, excluded or even discriminated against?	Have you had similar experiences when interacting with the healthcare system?
	Have there been situations in your life, for example in school or on the labour market, where you felt you had been in a privileged position compared to others?	Have you had similar experiences when interacting with the healthcare system?
Social inclusion and involvement	Are you involved in any social, political or charitable organisations?	Would you like to be involved? How?

### Data analysis

Interviews were transcribed verbatim with the help of the software f4© and pseudonymised to protect respondents’ identities. Transcription logs were written to record observations and initial reflections during the transcription process. The qualitative data analysis software MAXQDA© was used to manage the data, including interview logs, transcripts and transcriptions logs. A two-step approach to data analysis was applied: first, a random selection of 16 interviews with NAKO participants, corresponding to 20% of the dataset, was inductively coded for main themes and patterns (Braun and Clarke, 2006; also [7, 40] to develop the coding frame which was subsequently applied to the entire sample of participants. As the analysis progressed, identified themes were refined, condensed and abstracted. Such inductive thematic coding aims to avoid treating individual accounts as representative of social categories or groupings (also [5]). Interviews by non-participants were analysed separately and individually in a similar manner given the small dataset. Second, main themes were analysed deductively and compared across categories based on prior knowledge about significant intersections, namely sex/gender, age and self-reported migration status to identify differences and commonalities (see [5]). Social categories compared thus included self-reported men and women, age groups 18–39, 40–59 and 60 + , and people self-reporting as having a migration background and those not disclosing a migration background. For instance, inductively identified themes were queried as to whether and how sex/gender may inform such individual accounts, and how sex/gender interacts with other social categories. This step aimed to make connections between and across individual accounts, social categories and broader structural relations in order to make visible the factors and processes that shape participants’ attitudes towards study participation [36]. Observed variations were recorded using the memo function in MAXQDA©.

The coding frame and analytical procedure were developed by the first author and regularly discussed with the last author and independent researchers in data sessions at the authors’ institution to ensure quality and accuracy. Finding presented synthesise rationales both for and against participation as attitudes towards participation. Such attitudes are reported by main theme and, where meaningful differences according to social location were identified, these are subsequently described, mirroring the two-step procedure used in data analysis. Citations used are referenced by participants’ self-reported sex/gender and age.

### Ethics

Ethics approval for this study was granted by the institutional ethics committee of the Brandenburg Medical School Theodor Fontane.

## Results

### Sample characteristics

Overall, 80 individuals participated in our interview study. Of these 80 respondents, 67 had participated in at least the baseline assessment of the NAKO (some had additionally completed follow-up assessments) and 13 had declined the invitation to participate altogether. Of the overall sample, 39 identified as male while 41 identified as female. The age range of the study sample was 23 to 73 years. 49 respondents lived in stable, long-term relationships (married or co-habiting) and 56 had children. 71 were in regular employment (full time or part time) while 9 were not; of those not in regular employment, 2 were unemployed and 7 had retired (Table 2). Interviews were primarily conducted in person until March 2020; due to the Covid-19 pandemic, 26 interviews were conducted via telephone or video conference between February and April 2021. These differences had no discernible impact on the results; however, telephonic interviews were usually shorter in duration and no non-participants were recruited during this time.

**Table 2:** Sample description

Sex/gender	Participants		Non-participants	
	male	female	male	female
Age group				
18–39	9	12	1	3
40–59	17	13	2	4
60 +	9	7	1	2
Civil status				
single/divorced/widowed	10	15	2	4
married/in partnership	25	17	2	5
Children				
yes	26	20	3	7
no	9	12	1	2
In regular employment				
yes	28	23	4	8
no	6	9	0	1

### Attitudes towards participation

Thematic analysis revealed that respondents framed their attitudes towards participating in NAKO in predominantly four ways: wanting to make a contribution; seeking personalised health information; excitement and feeling chosen; and seeking social recognition, framing study participation as an opportunity for being ‘seen’. Apart from those merely citing logistical reasons, non-participants inhabited a different orientation towards scientific research due to adverse experiences with medicine or the healthcare system rather than framing it as a site of social distinction. Our intersectional examination of these themes found important nuances depending on respondents’ social locations according to sex/gender, age, migration status and social class; however, all rationales were inflected by wider social practices, experiences of marginalisation, forms of cultural capital, and culturally inflected understandings of science and health.

### Wanting to make a contribution

Respondents who had participated in NAKO predominantly framed their decision as a contribution to society as a whole, to the healthcare system or to scientific and biomedical progress (Table 3). Expressing gratitude for the opportunity to contribute to what they considered a central cause in the interest of society, study participation was enacted as a key avenue for shaping the future of this society. Also, study participation in NAKO was framed as a site at which societal tension or identification manifest, charging active engagement in these processes with moral meaning. Detailing their desire to contribute socially, respondents thus often likened study participation to other forms of societal or charitable involvement. They emphasised the centrality of such involvement as both a right and a responsibility they bear for a functioning democracy and social cohesion, also through participating in health research (Table 3).

**Table 3:** Attitudes towards participating in NAKO: main themes

Theme	Respondent characteristics	Quotes	Code(s)
Wanting to make a contribution	Participant, m, 27	‘I think it’s very important. It’s like the NAKO represented it, it is very important for society that we understand more about common risks and widespread diseases, how they arise etc.’	Societal significance
	Participant, m, 70	‘Well, it was important to me. You can always talk about society and complain about it, but one has to actively take part’	Societal significance
	Participant, f, 46	‘And with voting, it is similar, right? Many people around me say they don’t vote anymore… but I really think that I do have a responsibility, for society as a whole, not just for myself and my family… one is part of society, I expect something from it, but then I also have to give back to it’	Research participation as community work
	Participant, m, 54	‘Well, for me it is similar to, say, donating to the Third World. That I provide my time for important, interesting information to be collected’	Research participation as community work
	Participants, m, 53	‘I owe my life to allopathic medicine, at least two or three times…and thus I saw this as an opportunity to give a little bit back’	Desire to give back
	Participant, f, 55	‘I know this story from paediatric oncology… We were told, this was over 20 years ago, that data in paediatric oncology are centrally collected and doctors proceed according to specific therapeutic protocols [based on these data]. And so it was important for me, perhaps, to pay back something, to a certain extent, yes, to medicine, or to society’	Advancing science; desire to give back
Seeking personalised health information	Participant, f, 38	One reason, of course, was personal interest, you can get a check-up, you get medical tests and then the results, and perhaps you can see if something is not completely ok. I would not go to my GP out of boredom’	Rationales of study vs. healthcare system
	Participant, f, 48	‘Well, I had a small issue, nothing major, health-wise. And they conduct one or two examinations which normal GPs don’t do…’	Rationales of study vs. healthcare system
	Participant, f, 55	‘Well, but I still try to be physically active, within the realm of possibilities, and watch my diet etc.…and I didn’t expect the NAKO to uncover anything I didn’t already know about’	Participation as health practice
	Participant, f, 48	‘I think, I had done sports before and I still do sports, and regarding my diet, this is more – well I think there my health consciousness is increasing with age, right, and I’m really annoyed that I’m beginning to need reading glasses. I really hate it’	Participation as health practice
Excitement and feeling chosen	Participant, f, 46	‘Well I think it’s exciting, that I am part of it. Because from what I read in the information sheet, it is a very large study, Germany-wide, and I thought ‘why not’… I actually, oh, it was like ‘what, I was chosen by lot’, it was a bit like winning the lottery’	Being chosen; being part of something big
	Participant, m, 54	‘I don’t know anybody else who – maybe this also played a role. I was a chosen one, to participate (laughs)… First I was a bit suspicious, ‘why me’? But then I felt chosen and I wondered, how could I can reject it?’	Being chosen; sense of uniqueness
Using the study as a source of social recognition	Participant, f, 44	‘And it was a time in my life when many issues had cropped up for me, personal but also professional, and I thought ‘well, it’s actually a good moment, to look at this, for myself. So this was definitely involving self, self-interest. To participate and say, once I am there, I can also ask some questions’	Professional advice; time for oneself; being seen
	Participant, f, 73	‘I didn’t care at this point [that it took so long], one has to take some time for oneself. To get an independent assessment of "how did you manage this at the age of 70, that everything is OK"?’	Time for oneself; recognition of lifestyle; being seen
	Participant, f, 39	‘Well if I weighed, say, 125 kg and could barely leave my flat, I don’t think I would have gone… I think this has to do with the topic of recognition. "You’re on the right track, there are some issues but overall, it’s alright". Like an external affirmation of my lifestyle or something like that’	Recognition of lifestyle

For some, the desire to contribute was more directly expressed as a commitment to advancing medicine or the healthcare system, resulting from the confidence in medicine and other public institutions as forces of public good. This was especially the case for respondents who had positive experiences with these institutions, expressing their gratitude and desire to ‘give back’. These respondents foregrounded their indebtedness to the healthcare system which had created a social bond with the institutions of medicine and public health; they also evoked the self-expectation to be ever-conscious of repaying that debt. As such, the social glue that debt engenders shaped their sense of obligation and responsibility for medical research and the healthcare system.

Wanting to make a contribution was prevalent across our sample of NAKO participants. While those in the age group 40–59 with family or work constraints frequently cited logistical reasons for not being more actively involved in community work, they were apologetic of this perceived shortcoming, strongly supported such work and unanimously defended the significance of voting in general elections. Asked about any experiences of marginalisation or even discrimination, most explained they had not had such experiences. A notable exception were respondents with biographies in the former German Democratic Republic (GDR), old enough to have lived through so-called reunification (Table 4). These respondents described their sense of alienation and exclusion given the marginalisation of East German values, structures and political institutions during reunification [32]. Experiences of biographical rupture, the temporary loss of their livelihood and processes of othering (ibid.) conjured respondents’ professional and personal struggles. Here, the intersections of geographical/cultural origin and age overlapped in respondents’ experience, shaping their lack of affiliation with and disengagement from the institutions of the West German state. However, they eventually managed to re-establish professional careers, albeit often in lower-skilled professions, given their high social class, cultural resources, social networks and ability to adapt; their experience of marginalisation thus did not affect their active participation in health research and other public institutions long term.

**Table 4:** Key factors shaping rationales

Theme/ Code	Subtheme	Respondent characteristics	Quotes	Relevant intersections
Wanting to make a contribution	Experiences of marginalisation or discrimination	Participant, f, 55	‘Oh absolutely. During the time of the reunification, for example. As an East German. I’m actually a trained teacher. Worked in adult education. Had a diploma from [renowned university in Germany] and, this time, retrospectively, speaking of social disadvantage, this was a really, really awful time for us East Germans… I tried to remain in my profession, right? How some people were treating me!’	Geographical/cultural origin; age
	Experiences of marginalisation or discrimination	Participant, m, 70	‘Well I experienced this very actively, and I was really standing on the street and thought “what will become of you?”…You cannot imagine this. You’re sitting at your desk and someone, anyone, this was an awful time, someone who doesn’t understand *anything*…, comes in and says "get out of this room”’	Geographical/cultural origin; age
	Experiences of marginalisation or discrimination	Participant, f, 60	‘I was really curious…when I realised that this is not only about the indigenous German population but also about groups that have migrated to Germany… It’s clear that my fellow countrymen would need more treatment [than indigenous Germans], so I found the study really interesting’	Citizenship; social class; sex/gender; ethnicity
Wanting to make a contribution – advancing science	Positive evaluation of science, scientific literacy	Participant, f, 44	‘Every fairly educated person who went to college knows how difficult it is when the response rate is only 3%’	Social class; education; cultural capital
	Positive evaluation of science, scientific literacy	Participant, f, 45	‘Well, I think it’s always nice to have some participants, right? I think, during my studies, I also worked in market research and it’s always annoying to not have any, right?’	Social class; education; cultural capital
	Adverse experiences with science	Non-participant, f, 64	‘Well I have to start with the primordial soup. The primordial soup, for me, means, many years ago … simply to explain why I did say “oh no, not a study! Then they’ll find something again”’	Cultural capital, sex/gender, geographical/cultural origin
Seeking personalised health information	Care responsibilities	Participant, m, 54	‘Perhaps also due to my own children, I am more conscious about this [a healthy lifestyle]’	Sex/gender; age, marital status
	Care responsibilities	Participant, f, 39	‘Now that I have children, I think I also have a certain responsibility for being fit in the future’	Sex/gender; age, marital status
	Care responsibilities	Non-participant, f, 35	‘It was simply a question of time. At the time I was breastfeeding my son, he was about 5 months old… It was simply not an option to stay away for, I think it would have lasted about four hours’	Sex/gender; age, marital status
	Family responsibility	Participant, m, 65	‘Well, about a year and a half ago, I just spontaneously said ‘no, I won’t participate in this, what is this supposed to be?’ And then my wife said “oh, if I would get such an invitation, I would participate immediately”’	Sex/gender; age; marital status
	Family responsibility			
	Perceived vulnerability, biographical disruption	Participant, m, 65	‘I have always ruled this out. I have always said, “I’ll definitely stay healthy until I’m 85. Before I’m 85 I’m not going to the hospital, no way”. I’m still in top shape! And because I had this opinion of myself or this attitude, this diagnosis [prostate cancer] was really difficult for me’	Age; sex/gender, experiences of illness
	Perceived vulnerability, biographical disruption	Participant, f, 48	‘Well, perhaps this is associated with age, if you’re approaching 50… Previously, I had an image of myself, I had very few health-related problems, and now I had a few, smaller health problems, and then, when I was asked to participate in the study for the second time, well, I thought this is actually quite interesting’	Age; sex/gender, experiences of illness
Using the study as a source of social recognition	Juggling career and motherhood	Participant, f, 39	So this recognition from a second perspective, somehow. If I do this for myself, I’ll see “OK, it was good, it was bad”. But there [at the study centre] it’s written down somewhere or someone else says it… Maybe I also want to say that at the moment, while on parental leave, it is not always that easy to have these small, visible or acknowledged successes, or a sense of achievement…’	Gender; age; social class; marital status; sexuality

Experiences of exclusion were also raised by a middle-aged, female NAKO participant who had resettled to Germany after the dissolution of the Soviet Union. While she detailed experiences of exclusion and even physical abuse due to her ascribed ethnic origin as “Soviet” (she explicitly described her identity as Soviet rather than Russian), her story was also more complex. After losing her diplomas, including her PhD certificate and multiple awards from the government of the former Soviet Union, due to a personal tragedy, her identity as a highly educated and successful academic was shattered. Ascriptions of class, ethnicity, citizenship status and sex/gender intersected in leaving her marginalised and ultimately impoverished due to aggravating mental illness. These intersecting experiences of classism, nativism, sexism and inadequate mental health support did not stop her from participating in NAKO; however, she framed her reasons for participating not as contributions to society in general but as a way to render the representation of society by the NAKO more inclusive of ‘people like her’. Detailing interactions with her general practitioner who asked her to exercise more frequently after work, she sarcastically said: “doctor, *you* only commute between three rooms, but *I* commute between five jobs everyday […] *and* I have a daughter”, underlining her argument that healthcare and health research needed to be more inclusive of a range of experiences. Having identified the NAKO as an opportunity to be seen and to rectify broader processes of underrepresentation, her rationale not so much reflects a deep-seated confidence in public institutions but a willingness and ability to utilise them for her alternative vision of social progress (Table 4).

Respondents who aimed to specifically contribute to the advancement of science inhabited a strong cultural affinity to research across social locations, often working in academic or health-related positions themselves or with direct experience of conducting research (Table 4). They were well-versed in the practices of and barriers to research either through educational attainment, cultural affinity or first-hand experience, testament to their high social class status.

Indeed, it was in this orientation to research and medicine that we found the most significant contrast to non-participants in our interview sample. The majority of non-participants we interviewed chose not to participate in NAKO due to logistical reasons but would have participated for similar reasons under different circumstances. However, some explicitly advanced critical rationales against study participation and cited adverse experiences with or attitudes towards biomedicine and the healthcare system. For instance, one respondent told us of how her experience with the healthcare system as well as other social and political institutions had been shaped by the loss of her twins due to what she remembered as insufficient obstetric care in the GDR, as well as her attempts to divorce an abusive husband with little to no support from state social services during and after the divorce. This left her feeling abandoned, misunderstood and misdiagnosed by different actors in the healthcare system, stoking fears of yet another condition being discovered through the assessment at the study centre (Table 4). Other non-participants in our sample used their personal experience of pathologisation due to mental illness to advance larger claims about the insufficiencies of the healthcare system and the necessity for health policy and education, rather than research, to drive social change. Unlike participants in our sample, they challenged the implicit enactment of public health research as inherently good or socially progressive, foregrounding a theoretically-informed stance against biomedical epistemologies and their hierarchisation of other forms of truth-making. Some also abstained from voting, expressing a lack of agency and sense of alientation from broader social institutions.

### Seeking personalised health information

Another major motivation to participate in NAKO was the expectation of individual benefit in the form of personalised health information as health examinations were often perceived as a ‘check-up’ (Table 3). Precise reasons varied as to why participants desired personalised health information; some used the opportunity for a general assessment of their health not obtainable from the primary healthcare system. Others had a specific health-related concern they sought additional examination or advice for. Yet others sought a second opinion on a persistent health issue or due to logistical and time constraints, systemic barriers, or the dissatisfaction with their doctor-patient relationship. To them, study participation offered a unique chance to obtain high quality and comprehensive assessments, even though they were cognizant that these assessments would not lead to any diagnoses. Moreover, the longitudinal character of the study meant that they could track the development of their health, further fuelling their interest in taking part. For all but few who cited such personal benefit as motivating factor, study participation constituted a welcome addition to their health-seeking practices as they were actively engaged in maintaining and promoting their health.

While we did not find any meaningful differences across the social groups we compared (according to age, sex/gender and migration background) in terms of their interest in personalised health information per se, respondents did vary as to the reasons they were interested in such information in the first place. In particular, while they had been health-conscious before, respondents with young children had a heightened sense of responsibility for their own health, aiming to be role models (Table 4). While this was found for both men and women with young children when they assumed primary care responsibilities, the majority of respondents who reported this were women, reflecting broader societal patterns in the distribution of care work. Gendered care responsibilities also mattered in the narratives of older, male participants living in traditional, heterosexual marriages who emphasised the key role their wives occupied in their opting for healthier lifestyles. Here, a different caregiving role is at play: respondents’ partners did not act as role models but nudged them to adopt healthier practices, including their participation in NAKO, illustrating their responsibility for the well-being of the family (Table 4). At the same time, non-participants raised the lack of childcare as a deterring logistical factor, prohibiting them from participating in NAKO (Table 4); this illustrates the complex relations between care and family responsibility, social support and attitudes towards study participation.

Moreover, we found that experiences of vulnerability and biographical disruption associated with age, but also experiences of illness or accidents increased respondents’ interest in obtaining health information as these experiences had tarnished their self-perception as healthy, almost invincible. Put differently, embodied experiences of vulnerability and fallibility made respondents more perceptive to participate in NAKO in order to obtain longitudinal health data. While this theme was closely linked to the experience of aging, closer examination also revealed the gendered norms equating vulnerability with weakness and the feminine as younger, male participants provided more santitised and disembodied narratives of their bodily fallibility.

Non-participants did not inhabit such interests in obtaining personalised health information. Often, the very opposite was the case: as one respondent told us, she explicitly decided not to participate due to her concerns the study team would identify yet another risk factor for a chronic illness.

### Excitement and feeling chosen

Respondents were also motivated to join the study by a sense of excitement and curiosity; despite their precise understanding of random selection and statistical chance, they marvelled at having been selected, describing it as akin to ‘winning the lottery’. Popular depictions of the study as Germany’s largest had created a sense of uniqueness, codified by frequent use of the German “*auserwählt*” (chosen) rather than “*ausgewählt*” (selected). The use of religious language, equating the invitation to participate with a divine intervention, portrays the study as offering a kind of ontological security usually ascribed to religion, and an opportunity to take part in a collective project of salvation. As such, rather than the result of individual desire, the excitement and curiosity about the study was shaped by the cultural authority of science that prevailed among study participants across social locations: participants with fewer resources or forms of capital also displayed a high level of reverence that goes beyond mere trust, and believed in science’s ability (and inclination) to positively shape pressing social problems. Moreover, rather than merely the quality of examinations or specialised, high-tech medical equipment at the study centre, respondents characterised the source of their excitement as the opportunity of becoming part of ‘something big’ or special, inferring a sense of collective exclusivity. Non-participants who inhabited alternate orientations towards science did not experience such a calling but perhaps rather a *haunting* by science.

### Seeking social recognition

Participants also used the invitation as an opportunity to raise other personal queries, as ‘time for themselves’ and as a source of recognition and validation. This demonstrates the subjective value of an independent professional assertion of respondents’ health not in the form of biomedical results themselves but also through staff’s evaluation of and affective response to such results. Not least, it also illustrates the significance of the study as a site where participants feel they are being seen and heard, despite or perhaps precisely because of its large size and scope.

Our data exemplified the normative dimensions of such recognition for respondents’ self-perception as well as the intersections with respondents’ multiple social locations. For instance, one respondent explained that compared to completing cognitive exercises at home, at the study centre “it’s written down somewhere or someone else says it”, giving her “visible or acknowledged successes, or a sense of achievement” (Table 4). This was hard to come by, she explained, during her time on parental leave spent largely on caring for a small child. This and other examples speak to the all-encompassing and invisibilised responsibilities of motherhood, offering little immediate reward and challenging the respondents’ former sense of self as a high achieving academic. As such, the recognition offered by study participation is not only health-related, cognitive and social simultaneously; it may also be particularly appealing for highly educated women juggling the transition to motherhood.

The desire for recognition was also shaped by class, sex/gender, dominant body images and conceptions of health and responsibility. The same respondent cited above stated that “if I weighed, say, 125 kg and could barely leave my flat, I don’t think I would have gone” [to NAKO], equating self-worth with attractiveness-*cum*-thinness, responsibility and self-discipline for her health. Along with other such examples in our data, this points to a particularly pertinent image of (in-)adequacy in contemporary (German) culture that centres on health optimisation as the *sine qua non* of both individual and collective worth, shaped by prevalent and classed anxieties about feminity and obesity that may well cause stigma and shame in those not meeting this ideal.

## Discussion

This paper has analysed participants’ and non-participants’ attitudes towards participation in NAKO, illustrating that rationales for or against participation in longitudinal health research are part of a larger arsenal of social practices and contexts. For participants, the paper has documented the specific social practices wherein the production and circulation of longitudinal health data is not only cast as a non-negotiable good, but also as a form of civic contribution and a source of social recognition. Akin to what Davies et al. [18] have framed as ‘being part of the big picture’, participants cast population-based health research as a site of distinction where they can make a difference but also where they are being recognised as distinct or special. At the heart of this is a shared culture or value system that teleologically enacts health research as a universal force for societal and individual progress. While this discourse is also constructed by study personnel itself, this adds to the framing of population-based health research in the literature as tasked with improving population health and providing personalised health data, often framed as social versus individual benefits [19, 50]. Instead, our results illustrate that participation is not only part of general health practices but fulfils a range of social and cultural functions, spanning the obtainment of recognition, visibility and permissibility. This way, our findings also suggest that concerns over therapeutic misconception [55] may be unwarranted; respondents were well aware of the purpose of the research and the benefits of research procedures but identified a range of other benefits associated with study participation (also [69]). For non-participants, the paper has found that their decisions are not based on a lack of scientific literacy or disinterest but sometimes constitute an active defiance of dominant biomedical rationales and the values of health optimisation. As shown, while some drew on their own adverse experience with the healthcare system to inform their (very much reasonable) refusal to participate (also [3], others foregrounded their conceptually and theoretically-driven rationales against participation as a basis for staking claims for improving healthcare provision.

Our two-step, intersectionality-informed approach to data analysis has illustrated that most respondents shared this particular repertoire of values, attitudes, practices and collectively held ideas despite their diverse educational attainments, professional occupations and degrees of scientific literacy. These value systems and orientations even transgressed experiences of social exclusion and marginalisation: as described, the construction of health research as an arbitrator of much broader social questions extends, for some, to rectifying the societal underrepresentation of particular social experiences and groups, offering an exclusive opportunity to obtain recognition and raise concerns. In the case of East German participants, for instance, the cultural authority of public health research had triumphed over their political and social marginalisation, rendering it a unifying force despite respondents’ experiences of humiliation and biographical rupture. Similarly, the female respondent with self-ascribed Soviet origins used the study as a site for being seen and raising her own concerns. These examples challenge the often stereotypical and pathologising representation of particular groups as passive or disinterested, foregrounding their very informed decision-making in spite of prior adverse experience. Moreover, these examples illustrate the broader social, political and historical contexts that influence subjective experience and decision-making rather than locating (dis-)interest in research participation in particular groups themselves. Not least, they foreground intra-categorical intersections and reveal novel intersections previously not analysed to produce a more complex and nuanced account of social location and experience.

Gendered and classed patterns also strongly influenced rationales for or against study participation. For example, our analysis showed that the interest in obtaining personalised health data and the desire for social recognition is strongly shaped by family responsibility and gendered patterns of care work. Not surprisingly, predominantly female respondents, especially those with young children, raised this issue, exemplifying the gender care gap which, for Germany, found that women still perform 52.5% more unpaid care work than men, rising to 83.8% for couples with children and even to 108.3% if only considering direct forms of care work, i.e. child care [43]. Key to this care gap is the sociocultural discourse that place mothers, and especially young mothers, at the forefront of responsibility for moulding the health practices of their children: motherhood is not an individual attribute or a natural quality but a social institution, shaped by the societal expectations, experiences and structures associated with being a mother [73]. The cultural value systems discussed above thus overlap with existing, often internalised, ideals of good motherhood (or indeed, *woman*hood) in which the participation in health research is but a logical extension of broader social practices deemed culturally legitimate. Research on mothers’ “foodwork” found that this plays out particularly strongly in highly educated mothers, often seeking to counterbalance societal concerns that maternal employment and the time poverty it causes impacts negatively on children’s health [75]. Indeed, we found it was particularly female participants of higher class status who juggled multiple professional and personal aspirations, ‘doing’ class and gender [65] through their participation in health research.

Such gendered and classed doings are also evident in the ‘bodyist’ [72] equation of beauty, thinness, responsibility and self-worth as respondents seek confirmation of their social, cultural and health-related value (cf. [48] through their participation in NAKO, performing permissibility as legitimate recipients of medical and social recognition. As the respondent cited above implied, she would not have participated in NAKO had she not conformed to existing norms around body weight and health, illustrating the function of health research as a mediator of social recognition *qua* health status. While this might, to some extent, explain the observed differences in health status between participants and non-participants [21], it also illustrates that health research constitutes a key site where identities are negotiated and reconstructed. The performance of particular social roles in and through health-related practices is thus central for understanding differential patterns of participation.

The analysis in this paper has therefore shown that attitudes towards participation in health research are contingent on a range of personal biographies, culturally sanctioned ideals and values, and shaped by the complex intersections of social location and experience. Our anti-categorical and comparative approach as well as our understanding of cultural processes as underlying *all* social behaviour [54], rather than simply as a prism for minority ethnic status or ‘hard-to-survey populations’ [42] makes visible the specific and situated practices informed by both oppression *and* privilege. Indeed, while some participants identified as migrants or narrated experiences of nativist exclusion, they fully participated in predominant value systems and discursive frames. Participants ‘othered’ [68] by processes of ableism or heterosexism mentioned experiences of marginalisation in the healthcare system or everyday life, but these did not profoundly impact on their attitudes towards study participation. Simultaneously, our anti-categorical approach made visible other, emergent social categories of significance, particularly the lived experiences of middle-aged respondents from the former GDR whose biographies were significantly ruptured by German unification and continued processes of othering within Germany [32]. While some decided to participate in NAKO nonetheless, others’ experiences had moulded a decidedly disinterested or even contentious stance towards health research and public institutions more generally, contingent on the social and cultural resources available to them. Our approach thus paints a more complex picture of the intersections of oppression and privilege in attitudes to research participation, and illuminates positions that often remain invisible. Defying any linear, causal explanation between social location and study participation, this prompts both epidemiology and intersectionality to take into consideration the nebulous and messy dynamics of power and social life [16].

### Limitations

Despite its large sample size, the low response rate indicates we were only able to recruit a specific group of participants already inclined to participate in research. Similarly, the NAKO non-participants we interviewed are unlikely to be representative of the large group of so-called non-contacts [28] who not only refused to participate in the study but also to give feedback on the reasons for their non-participation. The small sample of non-participants may limit the reliability of our data; however, our specific aim was to understand particular rationales guiding (non-)participation rather than excavating representative narratives such that this does not hamper the strength of our analysis per se. A larger sample of non-respondents might perhaps have generated different results, especially had different recruitment strategies been used. Indeed, sampling and recruitment were also limited, especially from an intersectional perspective; for instance, the focus on age and sex/gender stemmed from the a priori assumption, based on existing evidence on research participation in other German cohort studies (e.g. Hasselhorn et al., 2014), that these constitute categories of significance in this context. Moreover, age and sex/gender were often the only socio-demographic characteristics available from non-participants, which may have prevented the inclusion of a broader range of experiences. From an intersectionality-informed standpoint, this may not do justice to the complex understanding of social location, and fails to fulfil the expectation to include and ‘give voice’ to (other) marginalised perspectives. Moreover, given the random sampling strategy from population registries applied in NAKO, more progressive methods for intersectionality-informed sampling such as respondent-driven sampling or time-space-sampling [9] that allow reaching and/or oversampling more marginalised populations were not feasible. This was exacerbated by the means of invitation, i.e. sending written letters exclusively in German, known to be ineffective for reaching individuals with insufficient German language skills [63, 42, 62]. Despite justified critique of an ‘ethnic matching’ or ‘race'-of-interviewer-effects’ approach that assumes a one-dimensional and static approach to social location [27], pairing respondents and interviewers with the same country of origin, ethnicity or language has often been recommended to improve the inclusion of diverse communities [42]. Given both our intersectional approach that aims to consider multiple dimensions of social identity simultaneously but also because of logistical limitations, we did not deploy such an approach; this, however, may have reproduced the exclusion of marginalised positions whose embodied knowledge, experiences and value systems may well produce different results to those represented in this paper. Moving forward, sampling strategies explicitly designed to adhere to the tenets of intersectionality [9] should be considered; moreover, the consideration of complexity may have to be balanced with the strategic essentialisation of identity categories in order to improve representativeness.

Nonetheless, we believe there is merit in our approach to intersectionality from or at ‘the centre’ that makes visible hitherto unexamined positions of privilege and their relations to health research. In data analysis, our anti-categorical approach was useful in inductively distilling key patterns without recourse to established social categories, but it also prevented us from asking targeted questions about specific intersections that might have been of interest, for example about participants’ disability status or other, more specific experiences of discrimination. Indeed, opting for a “multigroup” (McCall, 2005: 1786) study with resulting heterogeneity of the sample meant we could not engage with specific patterns or intersections in-depth but rather present a broad overview of the key themes in the data.

## Conclusion

This paper has discussed primary materials from interviews with people who have been invited to participate in the German National Cohort, Germany’s largest cohort study, and have either accepted or declined this invitation, using an intersectionality-informed perspective. It has found that attitudes towards cohort study participation can be categorised in four broad themes: wanting to make a contribution, seeking personalised health information, excitement and feeling chosen, and using the study as a source of social recognition. Overall, we have found that for participants, health research constitutes a site of distinction in the sense of making a difference and being distinct or distinguishable. Our intersectionality-informed approach thereby illustrates that these attitudes are fundamentally shaped by wider social experiences of privilege and marginalisation, albeit in complex and non-linear ways. Rather than finding particular (intersectional) groups being more or less receptive to participate in the study, we have found that individuals with a range of backgrounds and experiences mobilise the study for their own, often non-medical, objectives. Not least, health research does not actively attract or recruit prefigured identities but is also a site at which (gendered, classed etc.) identities are actively produced.

Three key, interrelated conclusions for public health practice follow from this. First, the complexity of social practice and the performative nature of study participation challenges the understanding of particular populations as more or less disadvantaged, and as more or less inclined to participate in health research. Rather than constituting stable entities with particular properties, individual and collective identities are shaped through and in interaction with a particular study, often in unexpected or contradictory ways. Relatedly, this challenges the assumption underlying much intersectionality research that the classical trias of race, class and sex/gender inherently designates marginalised positions. This also means, second, that recruitment strategies targeting specific population groups based on socio-demographic criteria may miss important similarities between groups and fail to recognise others. Recruitment efforts should aim to better understand the social structures and dynamics that shape individuals’ decision-making across groups rather than singling out specific groups, risking to further contribute to their essentialisation. While inclusive strategies such as multi-lingual invitation letters or the use of community gatekeepers have been proven useful for recruiting ‘hard-to-survey populations’ (though such efforts have limited success if study personnel, questionnaires etc. remain monolingual), understanding people’s attitudes as embedded in much wider socio-cultural practices helps shape more meaningful interactions with the groups public health research aims to serve. And third, intersectionality aims not only to understand social heterogeneity but also to combat social injustice; for cohort study recruitment and retention strategies, it is not immediately clear what a more just approach might look like given the historically justified scepticism by marginalised groups towards research participation [3]. The liberal imperative equating inclusion and representation with justice becomes dangerous in a context where even access to basic health care is often a struggle for some groups [59]. As such, intersectionality-informed public health research, including our own, ought to engage in the ethical reflection on how results will contribute to ameliorating much broader social inequities.

## Data Availability

The dataset generated and analysed during the current study are not publicly available as individual privacy of respondents could be compromised but is available from the corresponding author on reasonable request.

## Abbreviations

GDR: German Democratic Republic
NAKO: NAKO Gesundheitsstudie (German National Cohort)
